# Time-Resolved Small-Angle X-ray Scattering Studies during the Aqueous Emulsion Polymerization of Methyl Methacrylate

**DOI:** 10.1021/acs.macromol.2c01801

**Published:** 2022-11-09

**Authors:** Adam Czajka, Peter A. Lovell, Steven P. Armes

**Affiliations:** † Department of Chemistry, University of Sheffield, Dainton Building, Brook Hill, Sheffield, South Yorkshire S3 7HF, United Kingdom; ‡ Department of Materials, School of Natural Sciences, The University of Manchester, Manchester M13 9PL, United Kingdom

## Abstract

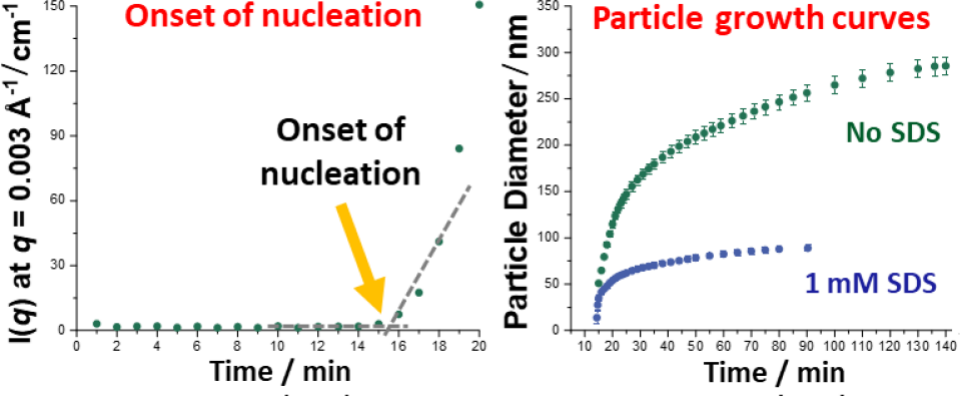

Recently, we reported time-resolved synchrotron small-angle X-ray scattering (TR-SAXS) studies during aqueous emulsion polymerization using a bespoke stirrable reaction cell (*J. Am. Chem. Soc.***2021**, *143*, 1474–1484). This proof-of-concept study utilized a semifluorinated specialty monomer (2,2,2-trifluoroethyl methacrylate) to ensure high X-ray contrast relative to water. Herein, we extend this approach to emulsion polymerization of methyl methacrylate (MMA) in the presence or absence of sodium dodecyl sulfate (SDS) at 70 °C. Solution conductivity measurements for this anionic surfactant indicated a critical micelle concentration (CMC) of 10.9 mM at this temperature. Thus, SDS was employed at either 1.0 or 20.0 mM, which corresponds to well below or well above its CMC. Postmortem analysis by ^1^H NMR spectroscopy indicated MMA conversions of 93–95% for these three formulations. We demonstrate that the X-ray contrast between water and PMMA is sufficiently large to produce high-quality scattering patterns during TR-SAXS experiments. Such patterns were fitted using a hard-sphere scattering model to monitor the evolution in particle diameter. This enabled (i) determination of the time point for the onset of nucleation and (ii) the evolution in particle size to be monitored during the MMA polymerization. The final particle diameters obtained from such TR-SAXS studies were consistent with postmortem DLS analyses, while TEM studies confirmed that near-monodisperse latex particles were formed. Micellar nucleation occurs within just 2 min when the SDS concentration is well above its CMC, resulting in a high particle number concentration and relatively small latex particles. In contrast, when SDS is either absent or present below its CMC, particle nuclei are formed by homogeneous nucleation over significantly longer time scales (14–15 min). In the latter case, adsorption of SDS onto nascent particles reduces their coagulation, giving rise to a larger number of smaller particles compared to the surfactant-free polymerization. However, the characteristic time required for the onset of nucleation is barely affected because this is mainly controlled by the kinetics of homogeneous polymerization of the relatively water-soluble MMA monomer within the aqueous phase. These results suggest that the aqueous emulsion polymerization of several other (meth)acrylic monomers, and perhaps also vinyl acetate, may be amenable to TR-SAXS studies.

## Introduction

Aqueous emulsion polymerization is a ubiquitous industrial process^1−10^ used by many chemical companies to manufacture around 10 million tonnes of polymer latex particles each year.^1,6,9^ Such formulations normally involve the polymerization of water-immiscible olefinic monomers in aqueous media using a water-soluble initiator and require efficient stirring to ensure formation of sufficiently small monomer droplets. Importantly, polymerization takes place predominantly within monomer-swollen latex particles. This compartmentalization of the polymerization within individual particles facilitates the efficient generation of high molecular weight polymer chains at high reaction rates with minimal change in the viscosity of the reaction mixture.^1,4−14^ Depending on their comonomer composition and particle size, the resulting latex particles have been widely used for many commercial applications,^1,2,4−9^ such as paints and coatings,^15^ varnishes,^16^ immunodiagnostic assays,^17^ concrete additives,^18^ or home and personal care products.^19−21^

Given its inherently heterogeneous nature, aqueous emulsion polymerization is much more difficult to monitor in situ compared to dispersion polymerization, which involves an initially homogeneous reaction mixture. Establishing the precise mechanism(s) operating during the relatively short particle nucleation period is particularly difficult.^1,4,6,9,22−24^ Nevertheless, we recently reported the first time-resolved small-angle X-ray scattering (TR-SAXS) study of an aqueous emulsion polymerization using a bespoke stirrable reaction cell.^25^ In our preliminary examination of the aqueous emulsion polymerization of styrene, we found that the X-ray contrast between polystyrene (density = 1.05 g cm^–3^) and water (density = 1.00 g cm^–3^) was too low for TR-SAXS experiments to be performed with sufficient temporal resolution. To circumvent this problem, we chose to study a semifluorinated specialty monomer, 2,2,2-trifluoroethyl methacrylate (TFEMA). The high density of PTFEMA homopolymer (1.47 g cm^–3^) leads to strong X-ray contrast relative to water and enables information-rich SAXS patterns to be obtained within a fraction of a second, which ensures excellent temporal resolution.

Herein we extend this proof-of-concept study to methyl methacrylate (MMA), which is an important commodity monomer. The aqueous emulsion polymerization of MMA has been widely studied over many decades^26−29^ and serves as an acceptable alternative to the well-established model system based on the aqueous emulsion polymerization of styrene.^1,4−6,9,10^ We show that the relatively high density of PMMA (1.18 g cm^–3^) enables high-quality SAXS patterns to be recorded on sufficiently short time scales to enable in situ studies of the aqueous emulsion polymerization of MMA either in the presence of an anionic surfactant or under surfactant-free conditions. It is perhaps also worth emphasizing that the aqueous solubility of MMA (15 g dm^**–3**^) is significantly higher than that of either styrene or TFEMA, so homogeneous nucleation^1,3−6,9,10,12,22,30,31^ is the dominant nucleation mechanism during its aqueous emulsion polymerization.

The kinetics of emulsion polymerization comprises three distinct time periods: Interval I, which involves particle nucleation and an increasing rate of polymerization; Interval II, whereby particle growth occurs at an approximately constant rate of polymerization and monomer droplets still exist; Interval III, in which particle growth occurs at a progressively slower rate of polymerization in the absence of any monomer droplets.^1,4−6,9−14,32,33^ Initiation of the polymerization involves monomer dissolved in the aqueous phase, even for monomers of very limited water solubility. This produces oligomeric radicals that grow to a critical degree of polymerization (a so-called *z*-mer). At this point, they become surface-active and enter monomer-swollen surfactant micelles and/or latex particles.^34^ These oligomeric radicals can also continue to propagate within the aqueous phase until a second critical degree of polymerization (a so-called *j*-mer) is attained, at which point they become water-soluble and undergo phase separation. At any point during their growth from a *z*-mer to a *j*-mer, such oligomeric radicals may enter either a monomer-swollen micelle or a latex particle. In the absence of any surfactant, nucleation proceeds by growth of oligomeric radicals to produce *j*-mers, which then undergo phase separation to form primary particles (i.e., single-chain nascent particles); this is homogeneous nucleation. Such primary particles exhibit poor colloidal stability even when using ionic initiators such as K_2_S_2_O_8_. This is because they carry just a single charge located at the chain end and hence undergo limited coagulation with each other until colloidal stability is attained, at which point nascent latex particles are obtained.^1,3−6,9,10,12,24,30,31,35−37^ Under such conditions, relatively large particles are produced. The presence of surfactant can significantly affect the nucleation mechanism and the final particle size. If the surfactant is present below its CMC, the surfactant can adsorb onto the nascent particles and reduce the extent of limited coagulation of the primary particles. If the surfactant is present above its CMC, then the dominant locus for particle nucleation tends to be the entry of oligomeric radicals into monomer-swollen micelles, which then immediately become nascent latex particles (micellar nucleation). Regardless of the mechanism, Interval I ends when nucleation is complete and monomer diffuses rapidly from the micrometer-sized monomer droplets through the aqueous phase to continuously replenish the monomer that is consumed by polymerization within the particles. This particle growth stage corresponds to Interval II and proceeds with a nominally constant monomer concentration within the particles. Eventually there are no remaining monomer droplets, and all of the unreacted monomer is located within swollen latex particles, which marks the onset of Interval III. During this final stage, polymerization proceeds at a progressively slower rate as the remaining monomer is gradually consumed without replenishment.

**Scheme 1:**
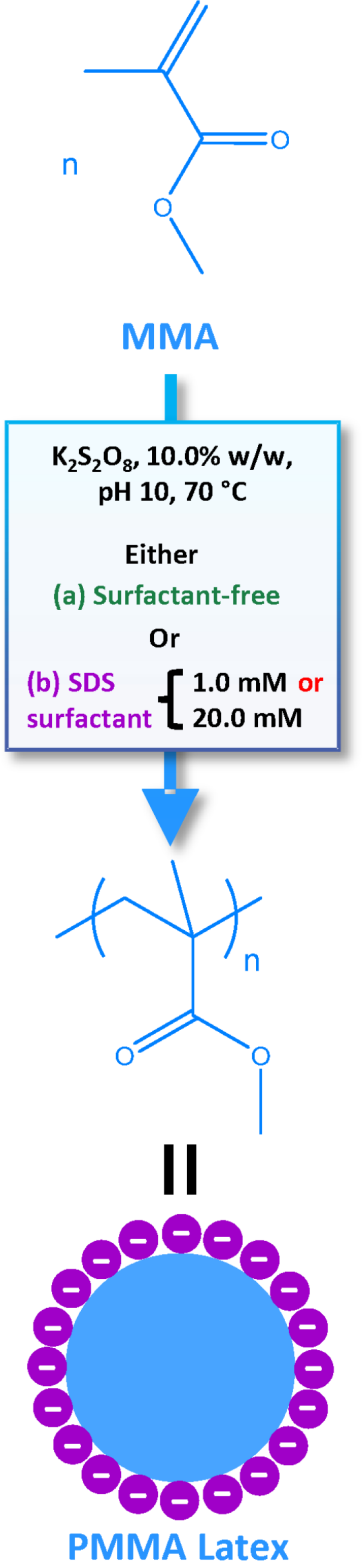
Schematic Representation of the Synthesis of PMMA Latex Particles via Aqueous Emulsion Polymerization of Methyl Methacrylate (MMA) Using an Anionic Free Radical Initiator (potassium persulfate, K_2_S_2_O_8_) at 70 °C Targeting 10% w/w Solids Either in the Presence of an Anionic Surfactant (SDS) or under Surfactant-Free Conditions

To the best of our knowledge, the effect of varying the surfactant concentration during aqueous emulsion polymerization has not yet been studied by TR-SAXS. This is no doubt because efficient stirring is essential to generate the micrometer-sized monomer droplets that are required for aqueous emulsion polymerization; this is simply not feasible within the glass capillaries commonly used for SAXS experiments. Fortunately, this technical problem can be resolved by employing a stirrable reaction cell, in which a capillary is placed immediately above the stirred reaction mixture (see Figure S1). However, in our recent proof-of-concept study of the aqueous emulsion polymerization of TFEMA, only a *single* surfactant concentration was investigated.^25^ Herein, we examine the effect of varying the surfactant concentration both well below and well above the CMC for the more industrially relevant emulsion polymerization of MMA using a model anionic surfactant, sodium dodecyl sulfate (SDS). More specifically, we conduct in situ SAXS studies during the emulsion polymerization of MMA at 70 °C at an SDS concentration of 0, 1.0 or 20.0 mM when targeting a final latex concentration of 10% w/w solids, see Scheme 1.

## Results and Discussion

### Determining the CMC of SDS at 70 °C

The surfactant concentration was selected to enable the aqueous emulsion polymerization of MMA to be conducted either above or below the CMC of the SDS surfactant, which is approximately 8.2 mM in aqueous solution at 25 °C.^38^ However, given that the MMA was to be polymerized at 70 °C, the CMC of SDS was determined at this temperature by in situ solution conductivity measurements, see Figure S2. Below the CMC, increasing the SDS concentration leads to a higher solution conductivity because there are additional ionic species (surfactant molecules) within the aqueous phase. However, micellization occurs when the SDS concentration exceeds the CMC, so most of the surfactant molecules no longer contribute to the concentration-dependent solution conductivity. Indeed, the CMC can be determined from the abrupt change in solution conductivity by taking the second derivative of such a data set, see Figure S2. Accordingly, the CMC for SDS at 70 °C was determined to be 10.9 mM.^38^ For an anionic surfactant such as SDS, a higher CMC is typically observed at higher temperatures because micellar self-assembly becomes less favorable under such conditions.^39^ Similar measurements conducted at 30 °C for an aqueous solution of SDS containing KPS (at a concentration relevant to that used in these polymerizations) and MMA (at its aqueous saturation concentration) indicated that the CMC (11.0 mM) was only slightly higher than that observed in the absence of KPS and MMA (8.2 mM). Hence, the three aqueous SDS concentrations studied herein (i.e., 0, 1.0, and 20.0 mM) provide an opportunity to explore particle nucleation and growth both in the absence of surfactant and for SDS concentrations either below or above the CMC of SDS at 70 °C.

### In Situ SAXS Studies during MMA Polymerization

SAXS is a well-established analytical technique in colloid and polymer science that offers unparalleled structural characterization.^25,40−54^ Moreover, synchrotron X-ray sources offer superb temporal resolution that enable the evolution of structure to be monitored in real time during chemical reactions. For example, in situ SAXS has been recently employed to monitor the various morphological transitions of block copolymer nano-objects that occur during their synthesis via polymerization-induced self-assembly (PISA).^55−58^

**Figure 1:**
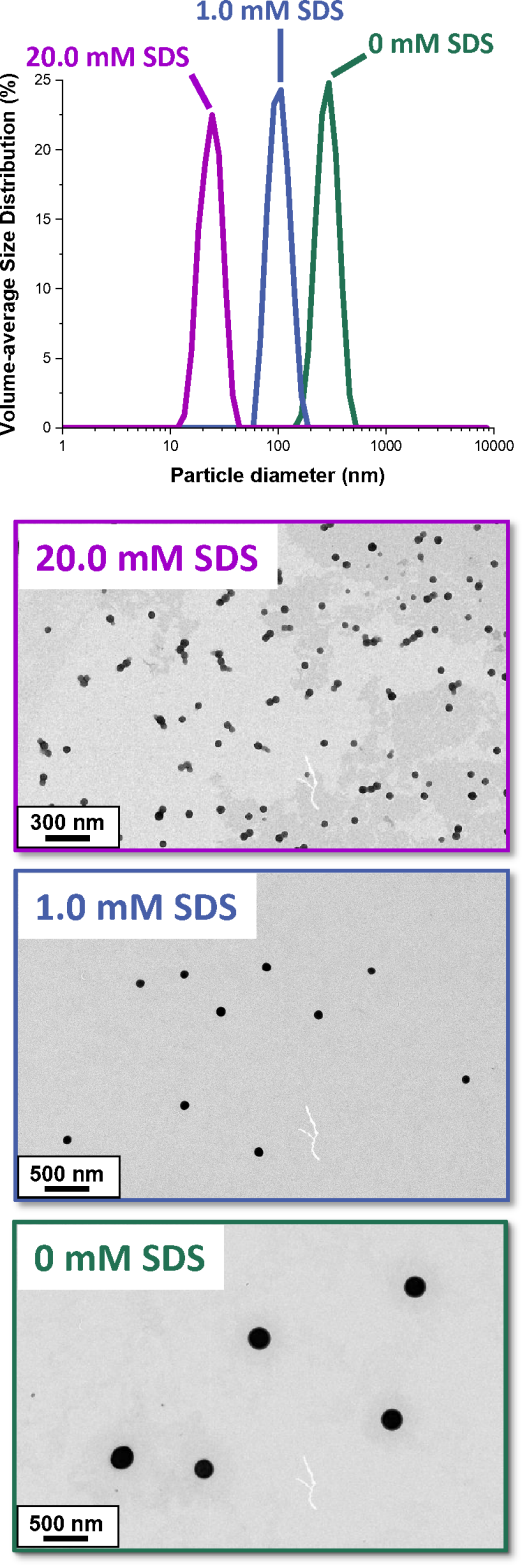
Postmortem DLS volume-average size distributions and corresponding TEM images obtained for the aqueous emulsion polymerization of MMA conducted using a stirrable reaction cell (see Figure S1) at 70 °C when targeting 10% w/w solids and varying the concentration of SDS surfactant as indicated.

In 2021, we reported the first in situ SAXS study during conventional aqueous emulsion polymerization.^25^ Using the stirrable reaction cell shown in Figure S1, the evolution in particle size was monitored during the aqueous emulsion polymerization of TFEMA.^25^ This cell has a total reaction volume of approximately 2.0 mL, which is sufficient to accommodate a small magnetic flea. This enables efficient stirring of the reaction mixture, which generates the micrometer-sized monomer droplets required for successful aqueous emulsion polymerization. The cell is heated to 70 °C via a circulating water jacket. SAXS patterns are then recorded at frequent intervals during polymerization, thus providing useful information regarding both nucleation and subsequent particle growth. As discussed above, we chose to study TFEMA simply because it provides strong X-ray contrast relative to water. Herein, we use the same experimental setup to perform TR-SAXS studies during the aqueous emulsion polymerization of MMA, which also provides sufficient X-ray contrast but is a much more industrially relevant commodity monomer. Thus, these new studies should be of broad interest. Importantly, the volume of the solution within the reaction cell is sufficient to enable postmortem analysis of the final reaction mixture using NMR, DLS, and TEM, see Table 1. ^1^H NMR spectroscopy analysis confirmed that at least 93% MMA conversion was achieved for all three formulations. The close agreement between the particle diameters reported by SAXS, DLS and TEM indicate relatively narrow particle size distributions, which demonstrates that particle nucleation was complete within a relatively short time scale in each case. Figure 1 shows the postmortem volume-average size distributions and corresponding TEM images, which confirm the formation of near-monodisperse spherical latex particles in each case. These results confirm that the stirrable reaction cell provides the efficient mixing that is a prerequisite for successful emulsion polymerization. According to the TR-SAXS data, the volume-average particle diameter is reduced from 284 to 89 to 24 nm when employing an SDS concentration of 0, 1.0 or 20.0 mM, respectively. Smaller latex particles are expected when using a higher surfactant concentration because physical adsorption of the anionic SDS molecules at the latex surface confers significantly higher surface charge than that provided by the sulfate end-groups derived from the KPS initiator. Final particle number concentrations (i.e., the number of latex particles per dm^3^ of the aqueous phase) and the particle surface area per SDS molecule have been calculated from the number-average particle diameters estimated by TEM and are summarized in Table 2. Clearly, the SDS surface coverage is far lower when latex particles are formed in the presence of SDS below its CMC. However, in both cases, the particle surface area per SDS molecule is significantly greater than the cross-sectional area of an SDS molecule (0.53 nm^2^).^6^ This suggests a relatively low surface coverage.

**Table 1:** Summary of Micellar Nucleation Times Determined from *I*(*q*) Plots, Final Particle Diameters Determined from Fits to SAXS Patterns Using a Sphere Model, Final Monomer Conversions Determined by ^1^H NMR Spectroscopy, and Postmortem Final Diameters Determined by DLS and TEM Analysis

			postmortem analysis		
aqueous SDS concentration (mM)	time for micellar nucleation^a^ (min)	final volume-average particle diameter^a^ (nm)	monomer conversion by ^1^H NMR spectroscopy (%)	volume-average particle diameter by DLS^b^ (nm)	number-average particle diameter by TEM (nm)
20.0	2	24 ± 1	95	28 (0.05)	26
1.0	14	89 ± 3	93	96 (0.02)	95
0	15	284 ± 10	93	292 (0.04)	285

a Determined by time-resolved SAXS studies (see main text for details of the calculations).

b Data given in parentheses are DLS polydispersities.

**Table 2:** Summary of the Final Particle Number Concentrations and Particle Surface Areas per SDS Molecule for the Aqueous Emulsion Polymerization of MMA at 70 °C When Targeting 10% w/w Solids in the Presence of 0, 1.0, or 20.0 mM SDS^a^

aqueous SDS concentration (mM)	final particle number concentration^b^	normalized final particle number concentration	final particle surface area per SDS molecule^c^ (nm^2^)
20.0	1.0 × 10^19^	1310	1.79
1.0	2.1 × 10^17^	27	9.84
0	7.8 × 10^15^	1	not applicable

a See main text for further experimental details.

b Calculated from the final monomer conversion and TEM number-average particle diameter (see Table 1) assuming that all unreacted MMA is located within the latex particles at the end of reaction and simple additivity of volumes, taking the densities for MMA and PMMA to be 0.94 and 1.18 g cm^–3^, respectively.

c Calculated from the TEM number-average particle diameter and final particle number concentration by assuming that all SDS molecules are adsorbed at the surface of the latex particles.

**Figure 2:**
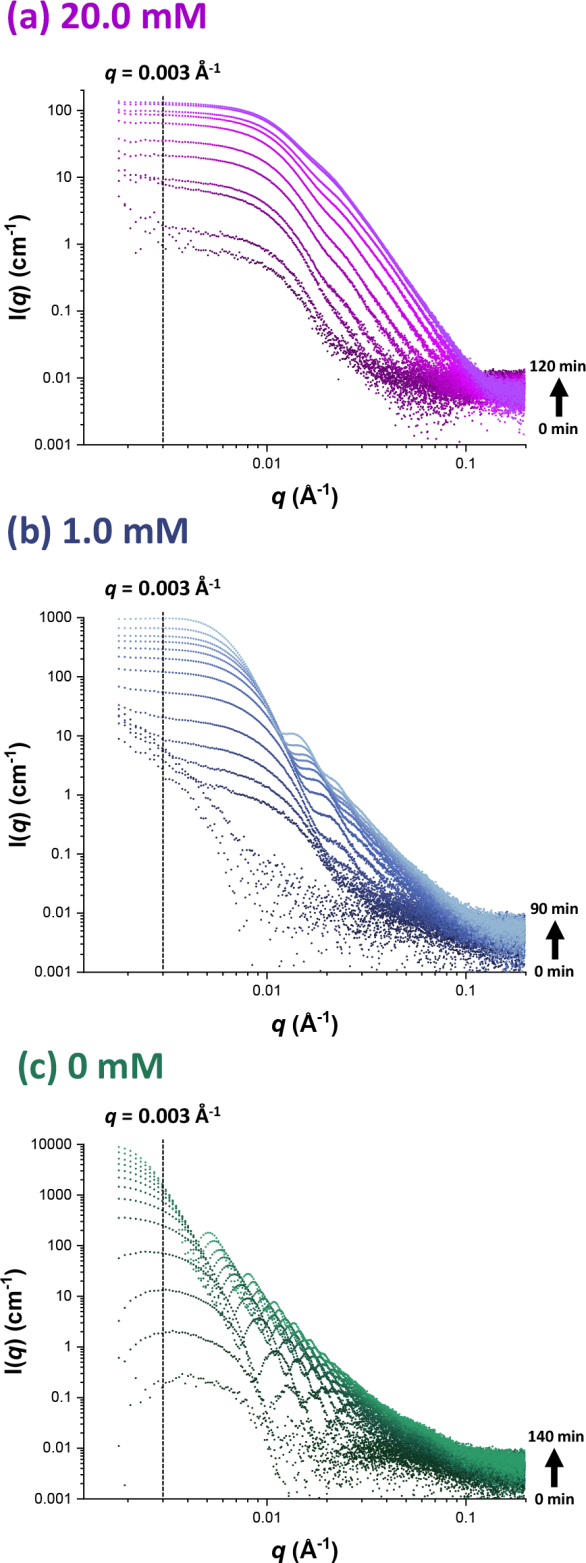
SAXS patterns recorded in situ during the aqueous emulsion polymerization of MMA at 70 °C when targeting 10% w/w solids using an SDS concentration of (a) 20.0, (b) 1.0, or (c) 0 mM.

Figure 2 shows the X-ray scattering intensity, *I*(*q*), plotted against the scattering vector, *q*, for selected SAXS patterns recorded during the aqueous emulsion polymerization of MMA at 70 °C for an SDS concentration of 0, 1.0 or 20.0 mM. More pronounced fringes are observed when using SDS below its CMC. This is consistent with the above observations that the particle size distributions become narrower under such conditions owing to the shorter nucleation period.^1,6,10,35^

### Onset of Particle Nucleation

**Figure 3:**
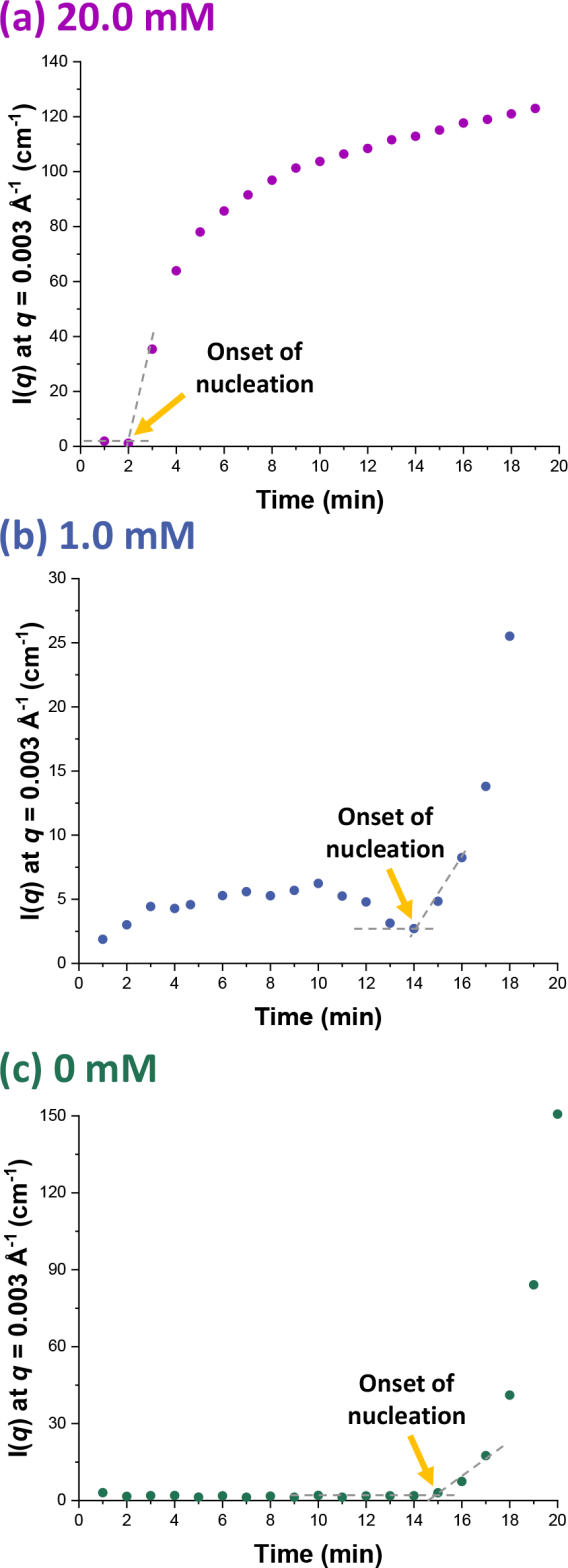
Evolution in *I*(*q*) recorded at an arbitrary *q* value (*q* = 0.003 Å^–1^) during the aqueous emulsion polymerization of MMA at 70 °C when targeting 10% w/w solids using an SDS concentration of (a) 20.0, (b) 1.0, or (c) 0 mM.

The volume of a scattering object is proportional to the scattering intensity, *I*(*q*), in the low *q* regime. Hence, measuring *I*(*q*) at an appropriate (fixed) *q* value can be used to identify the onset of nucleation, for which a significant upturn in *I*(*q*) is expected.^6,54,55^ In practice, this seemingly arbitrary *q* value should be chosen with some care so as to avoid local minima (or fringes), which would otherwise lead to undulations in the data. Inspecting Figure 2, we decided to select a *q* value of 0.003 Å^–1^. Figure 3 shows the variation in *I*(*q*) observed at this fixed *q* value for all three formulations. This reveals that the onset of nucleation occurred after approximately 2, 14 or 15 min for MMA polymerizations conducted using an SDS concentration of 0, 1.0 or 20.0 mM, respectively, see Table 1. Inhibition was minimized for these polymerizations by removing the inhibitor from the MMA monomer, thoroughly deoxygenating the reaction mixture using a nitrogen sparge for 30 min, and purging the stirrable reaction cell for 20 min with nitrogen gas just prior to use (see Supporting Information).

Early nucleation is expected when using a surfactant above its CMC because a very large number of monomer-swollen micelles are already present, which favors micellar nucleation. In this case, nucleation occurs as oligomeric radicals enter the monomer-swollen micelles after they have become *z*-mers. Thus, the SAXS data indicate that micellar nucleation is the predominant mechanism when using 20.0 mM SDS. In contrast, when using SDS below its CMC or for surfactant-free polymerizations, the growing radicals must grow much longer to become *j*-mers prior to undergoing phase separation from the aqueous phase to form primary particles. The rate of homogeneous polymerization within the aqueous phase is relatively slow owing to the limited aqueous solubility of the water-immiscible MMA monomer, and the rate of limited coagulation will be controlled nominally by the square of the concentration of primary particles; hence, the rate of particle formation is also relatively low.^1,4−6,9,30,31,37^ Thus the delayed particle nucleation observed in the absence of SDS or when using 1.0 mM SDS is consistent with the formation of colloidally stable nuclei via homogeneous nucleation, with limited coagulation of primary particles. There is only a relatively small difference between the nucleation onset times for these two polymerizations because the nucleation mechanism is essentially the same: the presence of SDS below its CMC simply leads to cessation of limited coagulation at an earlier stage owing to surfactant adsorption onto the nascent particle nuclei.

In situ monitoring of the solution conductivity during an aqueous emulsion polymerization enables the identification of Intervals I, II and III.^23,24^ For example, the boundary between Interval I and Interval II can be identified from the local minimum in the solution conductivity, while the local maximum corresponds to the Interval II/III boundary. Accordingly, we determined the solution conductivity during the aqueous emulsion polymerization of MMA in the presence of 20.0 mM SDS, see Figure S3. During Interval I, the concentration of free surfactant is reduced as nucleation occurs, which lowers the solution conductivity to a minimum value. According to Figure S3, the continuous reduction in solution conductivity that occurs after 1 min indicates a very early onset of nucleation. The minimum in conductivity observed after approximately 3 min indicates that nucleation is complete on this time scale. These observations are consistent with micellar nucleation and the onset of nucleation time scale of 2 min indicated by in situ SAXS studies for the same emulsion polymerization formulation (see Figure 3a).

### Particle Growth

**Figure 4:**
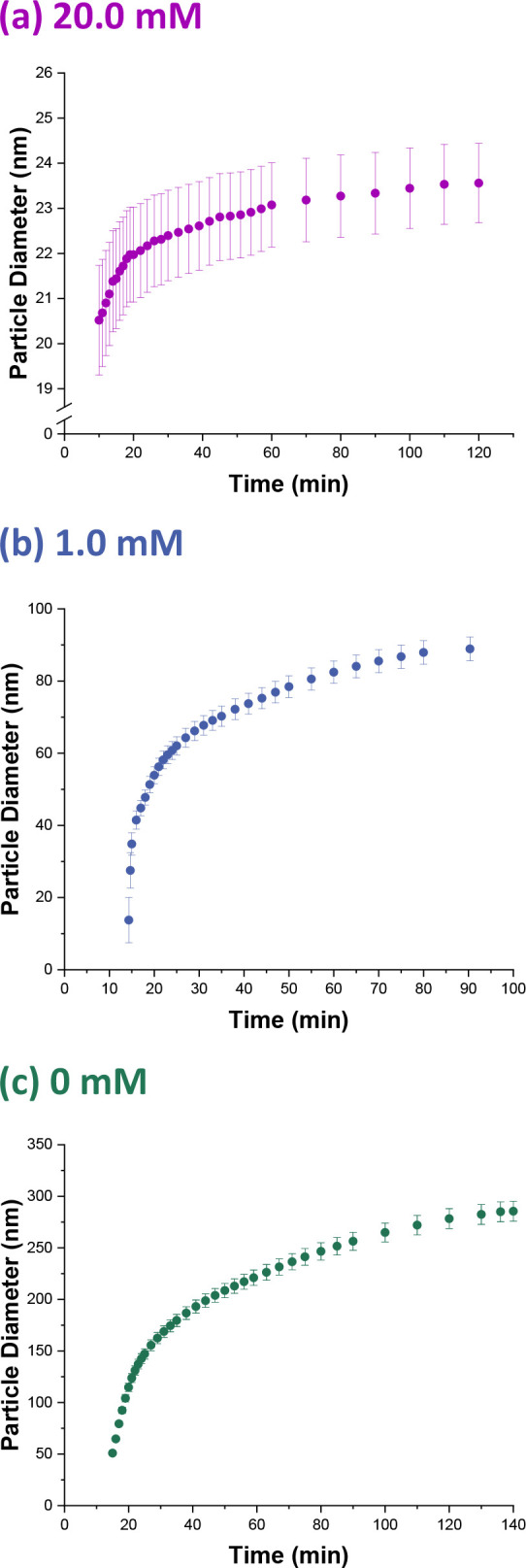
Evolution of the PMMA latex particle diameter over time determined by time-resolved SAXS studies conducted during the aqueous emulsion polymerization of PMMA at 70 °C targeting 10% w/w solids using an SDS concentration of (a) 20.0, (b) 1.0, or (c) 0 mM.

The scattering patterns shown in Figure 2 (which were recorded after the onset of nucleation) were fitted using a well-known scattering model for spheres.^59^ Because 10.0% w/w solids was targeted for each formulation, a hard-sphere structure factor (solved using the Percus–Yevick closure relation^60^) was incorporated to account for repulsive interactions between the highly anionic particles. Figure 4 shows the evolution in particle diameter for these three MMA polymerizations as determined by in situ SAXS studies using the stirrable reaction cell. For each formulation, rapid growth in particle diameter is observed immediately after the onset of nucleation but at a progressively slower rate of increase. This is normal for an aqueous emulsion polymerization because polymer is formed at a nominally constant rate during Interval II, and the particle diameter scales as the cube root of the particle volume. In the absence of any conversion vs time curves, it is not possible to comment further on the rate of polymerization. In principle, autoacceleration^61−63^ could cause an increase in the rate of polymerization during Interval III, which is predicted to begin at approximately 35% conversion (as calculated from the equilibrium concentration of MMA monomer within PMMA latex particles^27^ using MMA and PMMA densities of 0.94 and 1.18 g cm^–3^, respectively, and assuming that no change in volume occurs on mixing). However, given the relatively small size of the nascent nuclei (which are as small as 6 nm in diameter when using 2.0 mM SDS and around 51 nm in diameter in the absence of any SDS), heat transfer to the aqueous phase is expected to be very efficient, so any contribution from autoacceleration is most likely negligible in this case.

Eventually almost all of the MMA monomer is consumed, and the rate of polymerization tends to zero. In principle, the MMA polymerization may be judged to be essentially complete when there is no discernible difference between consecutive scattering patterns. However, the very small change in particle diameter that occurs during the final 10% of monomer conversion makes this a rather insensitive method. Instead, we emphasize that TR-SAXS can be used to monitor the evolution of particle diameter during MMA emulsion polymerization from the onset of particle nucleation and throughout the complete period of particle growth.

## Conclusions

The aqueous emulsion polymerization of MMA conducted in the presence or absence of a model anionic surfactant (SDS) has been studied by time-resolved synchrotron SAXS using a stirrable reaction cell. Postmortem analysis of the final latex particles by ^1^H NMR spectroscopy indicated MMA conversions of 93–95% for the three formulations studied. In situ SAXS and postmortem TEM and DLS analyses for the final particle diameter were self-consistent and confirmed that near-monodisperse spherical latex particles were formed in each case. In addition, the polymerization conducted well above the surfactant CMC was also monitored using in situ solution conductivity, with this technique indicating a characteristic time for the onset of particle nucleation that was in good agreement with that derived from the SAXS experiments.

We demonstrate that the experimental protocol recently employed for studying TFEMA emulsion polymerization by SAXS^25^ can be used to (i) determine the time scale for the onset of particle nucleation and (ii) monitor the evolution of particle diameter during MMA emulsion polymerizations throughout the particle growth stage. Data for the onset of particle nucleation are particularly informative. A micellar nucleation mechanism is predominant when SDS is present well above CMC and nascent particles are formed within 2 min; this results in a relatively high particle number concentration and hence very final small latex particles. On the other hand, when SDS is either absent or present well below its CMC, the onset of nucleation is significantly delayed and far fewer (and hence much larger) near-monodisperse latex particles are formed. This suggests that homogeneous nucleation occurs within a relatively short time scale, with limited coagulation of primary particles leading to the formation of colloidally stable particle nuclei. When used well below its CMC, SDS adsorbs onto nascent particles and reduces the extent of limited coagulation of the primary particles. This produces many more (and hence much smaller) particles compared to the surfactant-free polymerization. However, it does not significantly affect the characteristic time for the onset of nucleation, which is mainly controlled by the kinetics of polymerization of MMA within the aqueous phase. These findings confirm that increasing the surfactant concentration from zero to well above the CMC leads to a change in the particle nucleation mechanism during the aqueous emulsion polymerization of MMA.

Finally, we note that time-resolved synchrotron SAXS studies in conjunction with a stirrable reaction cell should enable studies of the aqueous emulsion polymerization of several other (meth)acrylic monomers and perhaps also vinyl acetate, since the corresponding homopolymers have comparable densities to that of PMMA. In principle, integration of a Raman or near-infrared spectroscopy probe into the reaction cell design should enable concomitant measurement of the instantaneous monomer conversion.^64^ Such an approach would offer even greater potential for identifying and understanding the various reaction mechanisms that can operate during aqueous emulsion polymerization. However, it remains to be seen whether the same approach can be extended to include more commercially relevant copolymerization formulations, such as the statistical copolymerization of methacrylates with acrylates that is widely used for the industrial manufacture of film-forming latex paints.

## Supporting Information Available

Full experimental details for all aqueous emulsion polymerizations and characterization protocols; solution conductivity data and plots; details of DLS data analysis, and the hard-sphere scattering model used for the SAXS analysis of the PMMA particles (PDF)
