# Supplementary material for: Time-Resolved Small-Angle X-ray Scattering Studies during the Aqueous Emulsion Polymerization of Methyl Methacrylate
Source: Macromolecules. 2022 Nov 9;55(22):10188–96. doi: 10.1021/acs.macromol.2c01801 (PMC9686128; doi:10.1021/acs.macromol.2c01801)
Supplement: Supplementary file 1 — ma2c01801_si_001.pdf [file ma2c01801_si_001.pdf]

Supporting Information for

Time-Resolved Small-Angle X-ray Scattering Studies During the Aqueous Emulsion Polymerization of Methyl Methacrylate

Adam Czajka, Peter A. Lovell and Steven P. Armes*

1. Experimental Section

Materials

Methyl methacrylate (MMA; 99%, Sigma-Aldrich,) was passed through a MEHQ inhibitor removal column prior to use. Sodium dodecyl sulfate (SDS, 99%), KPS (99%), $MgSO_4$ (98%), NaOH (98%), uranyl formate (0.75 % w/w) and $CDCl_3$ (99.8%) were purchased from Sigma-Aldrich (UK) and were used without further purification. Deionized water was obtained from an Elga DV25 water purifier.

In situ SAXS studies of PMMA latex particles using the stirrable reaction cell

The experimental protocol employed for the synthesis of surfactant-stabilized PMMA latex particles at an aqueous SDS concentration of 1.0 mM was as follows. KPS initiator (0.50 mg), SDS (0.60 mg) and deionized water (1.92 mL) were weighed into a 14 mL glass sample vial. The reaction mixture was adjusted to pH 10 by addition of 5 µL of a 0.1 M NaOH solution and then deoxygenated with N_2 gas for approximately 30 min. After removing its MEHQ inhibitor, MMA was deoxygenated separately using N_2 gas for 30 min with the aid of an ice bath. Deoxygenated MMA (0.21 g) was then added to the reaction mixture, which was transferred via degassed syringe to the stirrable reaction cell (containing a magnetic flea and equipped with a magnetic stirrer unit), which had been separately purged with N_2 gas for 20 min. This cell was then attached to the sample stage in station I22 at Diamond Light Source and aligned relative to the synchrotron SAXS beam. MMA polymerization was initiated by using a water-circulating jacket to heat the cell up to 70 ^{o}C as the X-ray beam shutter was opened. The polymerization was monitored until no further evolution in the 1D SAXS pattern was observed, at which point the reaction was assumed to be complete. This protocol was repeated using an aqueous SDS concentration of 20.0 mM. For *in situ* SAXS studies of surfactant-free charge-stabilized PMMA latex particles, the same synthesis protocol was adopted in the absence of

SDS using the following quantities and conditions: MMA (0.21 g), KPS (0.60 mg), deionized water (1.92 mL), 70 °C and pH 10.

In situ conductivity studies during the aqueous emulsion polymerization of MMA in the presence of SDS surfactant

The experimental protocol employed for determining the solution conductivity during the synthesis of SDS-stabilized PMMA latex particles at an aqueous SDS concentration of 20.0 mM was as follows. A 100 mL two-neck round-bottom flask was fitted with a Primo 5 conductivity probe. SDS (496 mg) and deionized water (74.8 mL) were weighed into this flask. The resulting reaction mixture was adjusted to pH 10 by adding 1 M NaOH solution (15 µL) via micropipet and then deoxygenating this aqueous solution with N_2 gas at 70 °C for approximately 30 min. KPS initiator (20 mg) and deionized water (2 mL) were weighed into a 14 mL glass sample vial and deoxygenated with N_2 gas for approximately 30 min. After removing its MEHQ inhibitor, cold MMA was deoxygenated separately using N_2 gas for 30 min with the aid of an ice bath. Degassed MMA (8.4 g) was added to the reaction mixture and stirred at 600 rpm for 3 min. The deoxygenated aqueous KPS solution was then added to the reaction mixture. The 'zero time' (t = 0 min) for this polymerization was taken to be the point at which the deoxygenated initiator solution was added to the reaction mixture. Conductivity data obtained using the Primo 5 probe were recorded using the video facility of a mobile phone mounted on a tripod. ^{1}H NMR spectroscopy studies indicated that a final MMA monomer conversion of 96 % was achieved.

1.1 Characterization

^{1}H NMR Spectroscopy

All NMR spectra were recorded in $CDCl_3$ at 298 K using a 400 MHz Bruker Avance-400 spectrometer (64 scans averaged per spectrum). MMA conversion was determined by comparing the integrated vinyl signals at 5.3 and 5.8 ppm to the signal assigned to PMMA at 3.7-4.0 ppm. A small amount of $MgSO_4$ was added to the NMR sample to remove the H_2O signal.

Dynamic Light Scattering (DLS)

DLS studies were conducted on 0.10% w/w aqueous dispersions at 25 °C in disposable plastic cuvettes using a Malvern Zetasizer NanoZS instrument that detects back-scattered light at an angle of 173°. Intensity-average hydrodynamic diameters were calculated via the Stokes−Einstein equation using a non-negative least squares (NNLS) algorithm. All data were

averaged over three consecutive runs. Intensity-average hydrodynamic diameters were converted into volume-average hydrodynamic diameters using Malvern Zetasizer Software (Version 7.01).

Transmission Electron Microscopy (TEM)

An aliquot extracted from each final reaction mixture was diluted one hundred-fold at 20 °C to generate 0.10% w/w dispersions. Copper/palladium TEM grids (Agar Scientific, UK) were surface-coated in-house to yield a thin film of amorphous carbon. The grids were then subjected to plasma glow discharge for 30 s to create a hydrophilic surface. Individual samples (0.10% w/w, 5.0 µL) were adsorbed onto the freshly-treated grids for 1 min and then blotted with filter paper to remove excess solution. To stain the particles, a 5.0 µL droplet of a 0.75% w/v aqueous uranyl formate solution was placed on the sample-loaded grid for 20 s and then carefully blotted to remove excess stain. The grids were dried using a vacuum hose. Imaging was performed using a Technai T12 Spirit instrument operating at 120 kV equipped with a Gatan 1 k CCD camera.

Small-Angle X-ray Scattering (SAXS)

SAXS patterns were recorded at a synchrotron facility (station I22 at Diamond Light Source, Didcot, Oxfordshire, UK). A monochromatic X-ray beam (λ = 0.124 nm), a 2D Pilatus 2M pixel detector (Dectris, Switzerland) and a q range of 0.02−2.00 nm^{-1} were used for these experiments, where $q = (4\pi \sin \theta)/\lambda$ corresponds to the modulus of the scattering vector and θ is half of the scattering angle. For these time-resolved measurements, a custom-designed stirrable reaction cell was used as the sample holder, see Figure S1. SAXS patterns were recorded every 10 seconds for 10 min, every 30 seconds for the following 30 min and every 60 seconds thereafter until no further change in consecutive SAXS patterns was observed. An exposure time of 100 ms was used for all frames collected. X-ray scattering data were reduced (integrated, normalized, and background-subtracted) using Dawn software supplied by Diamond Light Source. The X-ray scattering intensity for water was used for absolute scale calibration of the scattering patterns. Irena SAS macros for Igor Pro were used for modeling and further SAXS analysis.

2. Supporting Analysis

2.1 Determining volume-average particle sizes by DLS (Mie theory)

Instrument software (Malvern Zetasizer software version 7.11) was employed to calculate volume-average particle diameters from DLS data using Mie theory. A brief overview of how this calculation is performed within the software is provided below. For further information, see the Malvern website.[1]

According to Mie theory,[2] the scattering intensity measured by the photomultiplier detector can be described using the expression given below.

$$F(\theta,\Phi) = |S_1(\theta)|^2\sin^2\Phi + |S_2(\theta)|^2\cos^2\Phi$$

where θ and Φ correspond to the angles used to identify the location of the detector relative θ φ to the polarization plane of the incident light. S_1 and S_2 correspond to the scattering functions that embody the phase and amplitude of the scattered light. The Mie coefficients are contained within the scattering functions S_1 and S_2. Given the refractive index of the PMMA latex particles and the scattering angle, Mie theory can be used to calculate the scattering intensity as a function of particle diameter.

The number distribution can be calculated using the equation shown below, where I(x) corresponds to the DLS intensity distribution for a particle of diameter x, N(x) is the number distribution, and M(x, n_D, n_P) is the Mie scattering formula where n_D and n_P refer to the aqueous medium and particle respectively.

$$I(x) = N(x)M(x,n_D,n_P)$$

The volume distribution, V(x), can then be determined using the equation shown below, which assumes a spherical morphology.

$$V(x) = \frac{4}{3}\pi\left(\frac{x}{2}\right)^3$$

3. Supporting Figures

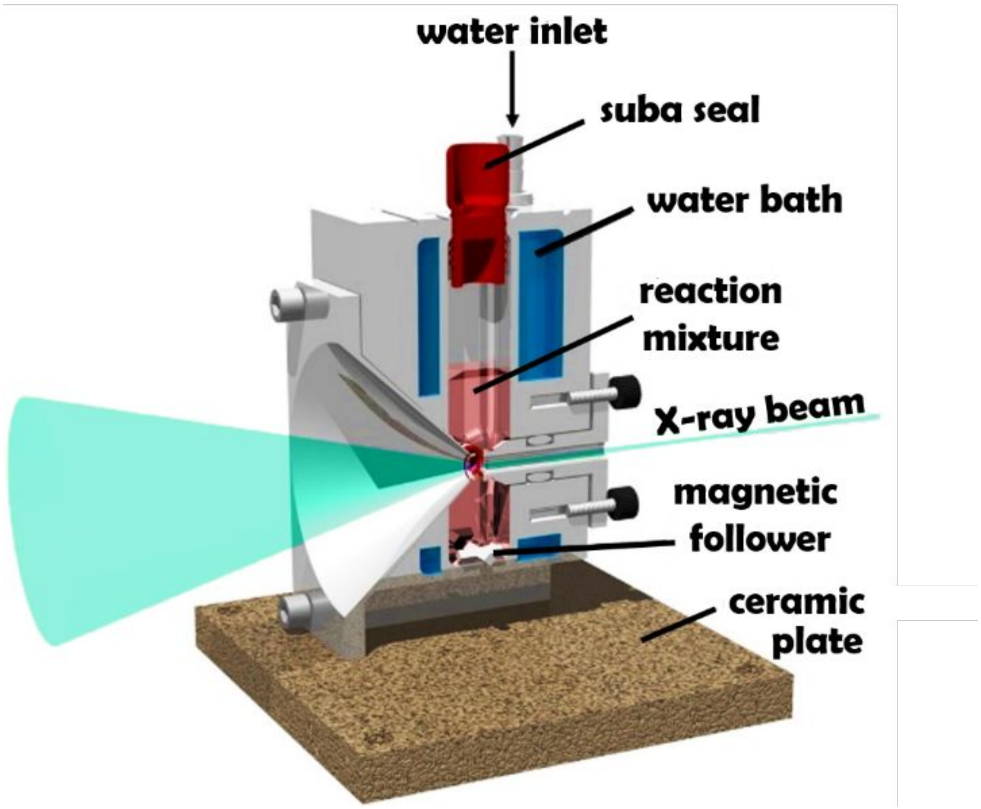

Figure S1. Schematic cartoon of the bespoke stirrable reaction cell used to conduct time-resolved SAXS studies during the aqueous emulsion polymerization of MMA conducted at 70 °C targeting 10.0% w/w either in the presence or absence of SDS surfactant.

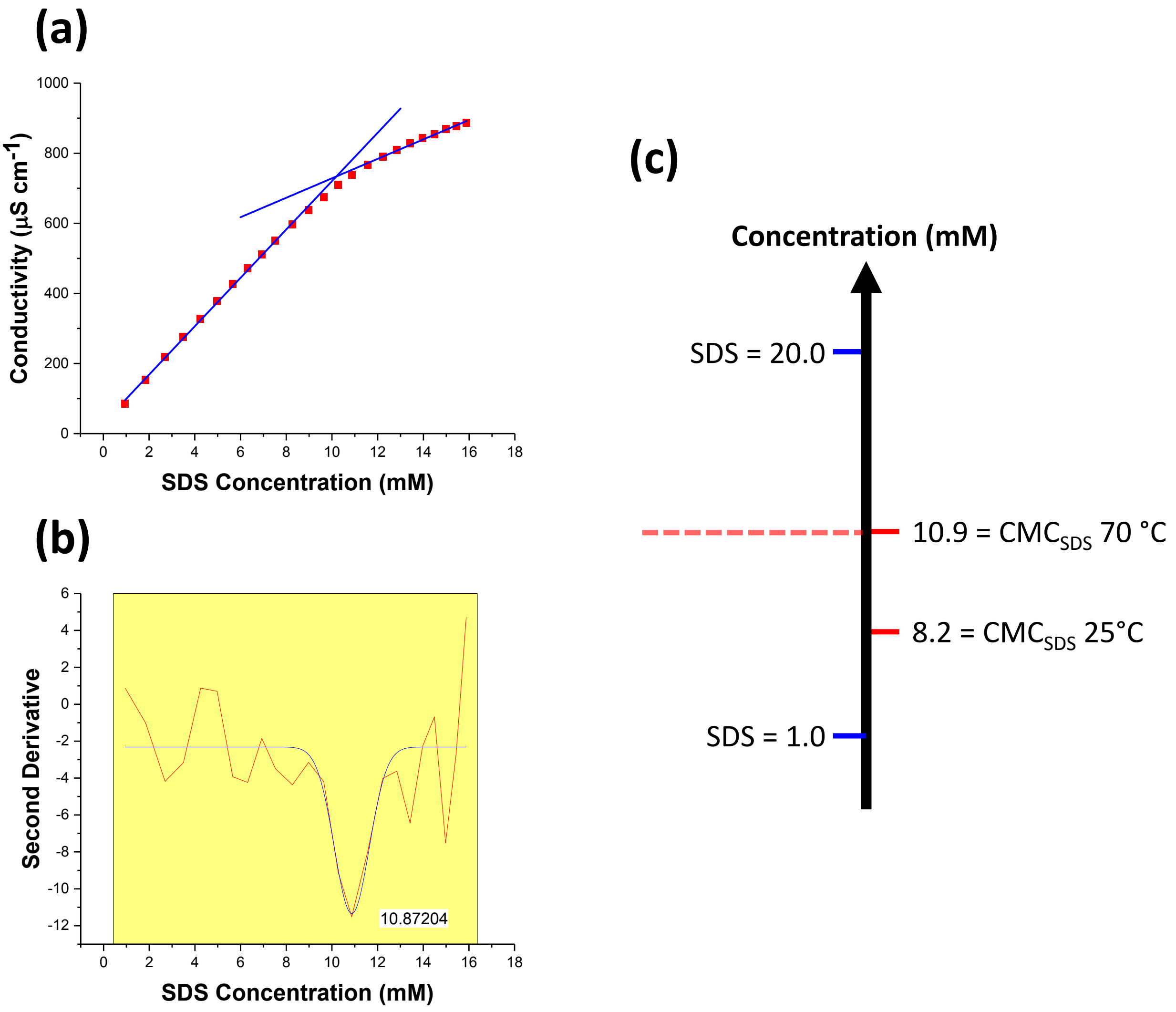

Figure S2. (a) Solution conductivity data obtained for a series of aqueous solutions of SDS at 70 °C. (b) The double derivative of the data shown in (a) with a Gaussian distribution applied to identify the critical micelle concentration (CMC). (c) The CMC determined for SDS at 70 and 25 °C and the SDS concentration for the three formulations studied herein (2.0, 0.1 and 0.0 mol% relative to MMA monomer).

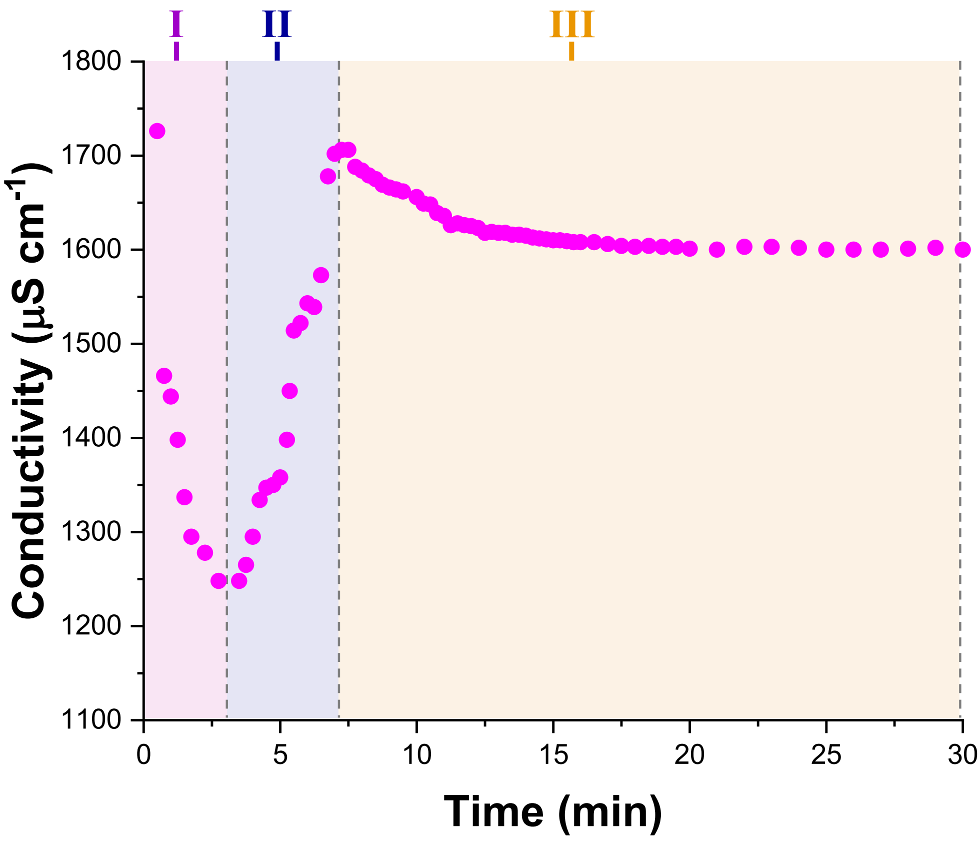

Figure S3. *In situ* conductivity data recorded during the aqueous emulsion polymerization of MMA at 70 °C targeting 10% w/w solids in the presence of 20.0 mM SDS. The three main time intervals (I, II and III) that are observed during aqueous emulsion polymerization are highlighted.

4. SAXS Model

The SAXS patterns produced by PMMA latex particles formed either (a) in the presence of SDS surfactant or (b) under surfactant-free conditions were fitted using a homogeneous sphere model and the Irena SAS macros for Igor Pro.[3]

The scattered intensity I(q) was measured as a function of the modulus of X-ray photon momentum transfer vector $q = (4\pi/\lambda)\sin\theta$, where 2θ is the scattering angle. The normalized scattered intensity (after subtraction of the solvent background) for a suspension of particles is given by:

$$\mathrm{I}(q) = \sum_{\mathrm{i}=1}^{n} S_{\mathrm{i}}(q) N_{\mathrm{i}} \int_0^{\infty} \left| F_{\mathrm{i}}^{\mathrm{pop}}(q,r) \right|^2 \Psi_{\mathrm{i}}(r)\, \mathrm{d}r \tag{S1}$$

where n is the number of different populations of particles in the suspension, $S_{\mathrm{i}}(q)$ is the structure factor arising from interparticle interactions, N_{i} is the number density of scattering particles of the ith population, $\left|F_{\mathrm{i}}^{\mathrm{pop}}(q,r)\right|$ is the form factor that describes the particle morphology (including contrast and volume parameters of the particles), and $\Psi_{\mathrm{i}}(r)$ is the size distribution function of scattering particles corresponding to the ith population. The PMMA latex particles can be represented by a single population of particles (i.e. n = 1 in eq S1) with the following functions and parameters describing the model:

$$F^{\mathrm{pop}}(q,r) = V(r)\Delta\xi f(q,r) \tag{S2}$$

where

$$f(q,r) = \frac{3[\sin(qr) - qr\cos(qr)]}{(qr)^3} \tag{S3}$$

is the form factor for a spherical particle,[65] $V(r) = {}^{4}/_{3}\pi(r + \Delta\mathrm{R})^3/3$ is the volume of the particles, and Δξ is the scattering contrast between the scattering length density of the latex

(ξ_{PMMA}) and the solvent (ξ_{water}). Here $\Psi(r)$ is the Gaussian (normal) particle size distribution such that:

$$\Psi(r) = \frac{1}{\sqrt{2\pi\sigma_{R_c}{}^2}} e^{-(r - R_c)^2/2\sigma_{R_c}{}^2} \tag{S4}$$

where R_c is the mean PMMA particle radius and σ_{R_c} is the standard deviation of the size polydispersity for the particle radius. Finally, N is given by

$$N = \frac{v}{\int_0^\infty V(r)\Psi(r)\,\mathrm{d}r} \tag{S5}$$

where v is the relative volume fraction of the PMMA particles in the aqueous dispersion.

Given the highly charged nature of these PMMA latex particles, a structure factor $S(q)$ that incorporates a hard-sphere structure factor solved with the Percus-Yevick closure relation is required to account for the interparticle interactions.[4]

In principle, monomer-swollen latex particles should exhibit a lower X-ray scattering contrast ($\Delta\xi$) compared to non-swollen latex particles, which would result in less intense X-ray scattering. In practice, the extent of monomer swelling is not expected to be high for this system and its effect on the X-ray scattering intensity is accordingly considered to be negligible.

References

1. https://www.materials-talks.com/wp-content/uploads/2017/01/FAQ-Calculating-volume-distributions-from-DLS-data.pdf
2. G. Mie, *Ann. Phys.*, 1908, **4**, 377.
3. J. Ilavsky and P. R. Jemian, *J. Appl. Crystallogr.,* 2009, **42**, 347-353.
4. A. Muratov, A. Moussaid, T. Narayanan and E. I. Kats, *J. Chem. Phys.,* 2009, **131**, 54902.
